# Impact of Chronic Exposure to Sublethal Doses of Glyphosate on Honey Bee Immunity, Gut Microbiota and Infection by Pathogens

**DOI:** 10.3390/microorganisms9040845

**Published:** 2021-04-15

**Authors:** Loreley Castelli, Sofía Balbuena, Belén Branchiccela, Pablo Zunino, Joanito Liberti, Philipp Engel, Karina Antúnez

**Affiliations:** 1 Laboratorio de Microbiología y Salud de las Abejas, Departamento de Microbiología, Instituto de Investigaciones Biológicas Clemente Estable (IIBCE), Avda, Italia 3318, Montevideo 11600, Uruguay; castelli.loreley@gmail.com (L.C.); sofiaf.balbuena@gmail.com (S.B.); pzunino@iibce.edu.uy (P.Z.); 2 Sección Apicultura, Instituto Nacional de Investigación Agropecuaria, Colonia 70002, Uruguay; bbranchiccela@inia.org.uy; 3 Department of Fundamental Microbiology, University of Lausanne, 1015 Lausanne, Switzerland; joanito.liberti@unil.ch (J.L.); philipp.engel@unil.ch (P.E.); 4 Department of Ecology and Evolution, University of Lausanne, 1015 Lausanne, Switzerland

**Keywords:** glyphosate, pesticides, honey bee gut microbiota, honey bee immune response, *Nosema ceranae*, deformed wing virus, honey bee health

## Abstract

Glyphosate is the most used pesticide around the world. Although different studies have evidenced its negative effect on honey bees, including detrimental impacts on behavior, cognitive, sensory and developmental abilities, its use continues to grow. Recent studies have shown that it also alters the composition of the honey bee gut microbiota. In this study we explored the impact of chronic exposure to sublethal doses of glyphosate on the honey bee gut microbiota and its effects on the immune response, infection by *Nosema ceranae* and Deformed wing virus (DWV) and honey bee survival. Glyphosate combined with *N. ceranae* infection altered the structure and composition of the honey bee gut microbiota, for example by decreasing the relative abundance of the core members *Snodgrassella alvi* and *Lactobacillus apis*. Glyphosate increased the expression of some immune genes, possibly representing a physiological response to mitigate its negative effects. However, this response was not sufficient to maintain honey bee health, as glyphosate promoted the replication of DWV and decreased the expression of *vitellogenin*, which were accompanied by a reduced life span. Infection by *N. ceranae* also alters honey bee immunity although no synergistic effect with glyphosate was observed. These results corroborate previous findings suggesting deleterious effects of widespread use of glyphosate on honey bee health, and they contribute to elucidate the physiological mechanisms underlying a global decline of pollination services.

## 1. Introduction

Honey bees (*Apis mellifera*) play an essential role in the environment, being important pollinators that contribute to agricultural production and the maintenance of natural ecosystems [1,2]. Besides that, honey bees produce honey, wax and propolis, among other products, which have been used for food, medicine and industrial purposes for centuries [2].

In recent years, large scale honey bee colony losses have been reported worldwide, which can reach 45% annually depending on the country [3,4,5,6]. Land use intensification, mainly the expansion of agricultural crops and the massive use of pesticides, together with the infections by multiple pests and pathogens, are among the most important causes of such losses [7,8]. While honey bees are not target organisms for most pesticides, they are exposed through collecting pollen and nectar from flowers and plant resins, and drinking water from rivers/lakes/ponds/etc. [9,10,11]. Pesticides are able to reach the hive and are commonly detected in honey and wax [11,12].

Glyphosate is the most used pesticide in the world [13]. It inhibits the enzyme 5-enolpyruvyl-shiquimate-3-phosphate synthase (EPSPS), an enzyme of the shikimate pathway that participates in the biosynthesis of aromatic amino acids and other secondary metabolites in plants, so it is widely used for weed control. Between 1974 and 2014, more than 8.6 billion kg of the active product were applied worldwide. Glyphosate use has increased by more than 15 times since 1996, due to the extended use of the product Roundup Ready™ (Monsanto) in genetically modified crops resistant to glyphosate [13]. Although different studies have reported the negative effect of glyphosate on honey bees, including detrimental effects on behavior, cognitive, sensory and developmental abilities (reviewed in [14]), its use is still allowed.

Recent studies have shown that glyphosate alters the structure and composition of the honey bee gut microbiota through the inhibition of the EPSPS enzyme, which can be coded by the genome of several microorganisms [15,16,17,18]. The gut microbiota plays an important role in honey bee metabolism, growth, development, immunity and defense against pathogens [19,20], so alterations of this community (dysbiosis) could have severe consequences on honey bee health.

The negative effect of pesticides could be enhanced by the interaction with pests and pathogens, such as the microsporidium *Nosema ceranae* [21]. This pathogen infects honey bees when they ingest food contaminated with spores, generating intestinal disorders, early aging and reducing the life-span of the bees. They negatively affect honey bee populations as they can kill entire colonies (reviewed in [21]). *Nosema ceranae* further weakens the bees’ immune response [22,23]. In addition to this microsporidium, more than 70 RNA viruses have been detected in honey bees [24]. Deformed wing virus (DWV) is one of the most studied honey bee viruses, since it, together with the *Varroa destructor* mite, has been associated with large scale colony losses [25,26]. Honey bees show different strategies to prevent infection by pathogens. Social immunity includes cooperative behaviors between individuals, such as grooming or hygienic behavior, while individual defenses include mechanical, physiological and immunological responses [27]. Doublet et al. [28], identified a set of genes that respond in a unified way to infection by different pathogens (*V. destructor*, *N. ceranae* and RNA viruses), including *hymenoptaecin, defensin, abaecin* and *lysozyme*, among others. However, recent studies have shown that some pesticides can affect the honey bee’s immune response [29,30] and facilitate the multiplication of pathogens [31]. As an example, Di Prisco et al. [31], demonstrated that the neonicotinoid insecticide clothianidin affects honey bee antiviral defenses and promotes the replication of the DWV.

In order to deepen in our understanding of the effects of pesticide exposure on bee health, we aimed to elucidate the impact of chronic exposure to sublethal doses of glyphosate on the honey bee gut microbiota, immune response, and survival, and its potential interaction with the widespread pathogens *N. ceranae* and DWV.

## 2. Materials and Methods

### 2.1. Chemicals and Solutions

Based on the glyphosate LD50 reported on the Pesticide Properties DataBase (100 μg per bee, [32]) and the realistic doses found in the field [33], the concentration 10 mg/L was chosen as the sublethal working dose. Glyphosate (active ingredient, Sigma-Aldrich, Taufkirchen, Germany) was administered in 50% sucrose syrup. It is estimated that the consumption of 10 μL of syrup per day generates the consumption of 0.10 g of glyphosate per bee per day (one thousand times less than its LD50). We prepared a stock solution of 20 mg/L, dissolving the active principle in sterile distilled water. We then diluted this solution in 50% sucrose syrup to a final concentration of 10 mg/L, and used it to feed the bees.

### 2.2. Spore Suspension of Nosema ceranae

A fresh spore suspension of *N. ceranae* was prepared from honey bees collected from a naturally infected colony belonging to a commercial apiary located in San José, Uruguay. Sample was collected by technicians from the Apiculture Section, Veterinary Laboratory Miguel Rubino, of the Ministry of Agriculture, Livestock and Fisheries (MGAP), and sent to our laboratory. Spores were collected using the centrifugation method as explained in the COLOSS Beebook [34]. The spores were quantified with a Neubauer chamber (hemocytometer) under an optical microscope at 400× magnification. The determination of the *Nosema* species was performed by multiplex PCR, as described by Martin-Hernández et al. [35]. Spores were maintained at room temperature, in the dark, and were used within one week of collection.

### 2.3. Experimental Design

We collected frames with sealed brood of *A. mellifera* (local hybrids between *Apis mellifera scutellata, Apis mellifera ligustica* and *Apis mellifera mellifera*) from three different colonies belonging to an experimental apiary located at the Veterinary School, Universidad de la República (UdelaR), Montevideo, Uruguay. The frames were incubated at the Department of Microbiology, IIBCE, with temperature and humidity mirroring natural conditions (Temp: 34 °C and Humidity: 60%). When bees began to emerge, we placed them into 12 cages, in groups of 70 bees per cage. These cages were then divided into four groups (in triplicates) and each group received a different treatment: bees were either (i) fed with 50% sucrose syrup contaminated with glyphosate (10 mg/L, G); (ii) fed with syrup containing *N. ceranae* spores (N); (iii) fed with syrup containing glyphosate (10 mg/L) and *N. ceranae* spores (GN); or (iv) fed only with syrup as a control (J).

Briefly, 24 h after emergence honey bees from groups N and GN were subjected to mass-inoculation with 1 mL of a fresh *N. ceranae* spores suspension in 50% sucrose syrup (estimate doses 100,000 spores per bee) [34] in graduate syringes, while honey bees from groups G and J received 1 mL of 50% sucrose syrup. Once the syrup was completely consumed (about 24 h later), honey bees were fed *ad libitum* with fresh sucrose syrup (groups J and N) or sucrose syrup contaminated with glyphosate (groups G and GN) using new graduate syringes. Every day we counted and removed dead individuals, estimated food consumption (determining the change in syrup volume in the graduated syringes), and provided the bees with freshly prepared food.

### 2.4. Bee Microbiota Analysis

– DNA extraction: After seven days of chronic exposure to glyphosate (or sucrose syrup), ten honey bees per treatment (three/four bees per cage from the three cages) were sampled to analyze their gut bacterial community. Honey bees were externally sterilized using a chlorine solution 1% [36]. Guts were extracted and individually homogenized in 500 μL of PBS using ceramic beads and a FastPrep-24™ during 40 s at 6.0 m/s. Then, samples were centrifuged for 1 min at 5000× *g* and the supernatants were transferred to sterile tubes. DNA was extracted from the supernatants using the SDS-CTAB method as previously reported [37]. Then, it was quantified using a NanoDrop1000 spectrophotometer (Thermo Scientific™, Waltham, MA, USA) and concentrations were normalized to 10 ng/μL.
– 16S rRNA amplicon sequencing: DNA was sent to Macrogen (Seoul, Korea) for library construction and 16S rRNA amplicon sequencing, as described by Illumina preparation guide (https://support.illumina.com/documents/documentation/chemistry_documentation/16s/16s-metagenomic-library-prep-guide-15044223-b.pdf, accessed on 31 July 2020). Briefly, sequencing libraries were built using a two-step PCR strategy. The V3-V4 region of the 16S rRNA gene was amplified using primers Bakt_341F (5′-CCTACGGGNGGCWGCAG-3) and Bakt_805R (5′-GACTACHVGGGTATCTAATCC-3′) with overhang adapters attached and 25 amplification cycles. Then, a second PCR was performed to attach dual indices and sequencing adapters using the Nextera XT Index Kit. In both cases, PCR conditions and programs recommended by Illumina preparation guide were used. Sequencing was carried out using Illumina MiSeq 2 × 300 bp.

### 2.5. Immune Gene Expression Analyses and DWV Quantification

– RNA extraction and cDNA synthesis: At 0, 7 and 14 days of chronic exposure to glyphosate (or sucrose syrup), we sampled twelve bees per treatment (four bees per cage, per three cages) to analyze the expression of genes associated with the immune response, as well as for the quantification of DWV, and stored them at −80 °C. Individual bees were homogenized in lysis buffer (Invitrogen) and subjected to RNA extraction using the Mini Kit PureLink RNA (Ambion, Carlsbad, CA, USA), according to the manufacturer’s instructions. One microliter of total RNA was treated with DNAse I (Invitrogen, Carlsbad, CA, USA) and used to generate first-strand cDNA using the High-Capacity cDNA Reverse Transcription kit (Applied Biosystem, Foster City, CA, USA), also according to the manufacturer’s instructions.
– qPCR: Relative expression of different genes was assessed by qPCR using previously reported primers. Genes included *lysozyme* [38], *glucose dehydrogenase* [38], *hymenoptaecin* [38], *vitellogenin* [39] and *prophenoloxidase* [MC-PPO-F: CGCAACTTAGATGAAAATAGACC and MC-PPO-R: TTGAGGCATCCTTACAACCA, Corona M., personal comm.]. We also quantified infection titers of DWV by qPCR [40]. Ribosomal protein S5 (RPS5) was used as a housekeeping gene to normalize the variation in the amounts of cDNA [41]. The reaction mix consisted of 1X Power SYBR^®^ Green Master Mix (Invitrogen), 0.3 μM of each primer, RNAse-free water and 2 μL of 1:10 diluted cDNA in a final volume of 20 μL. PCR reactions were carried out using a BIO-RAD CFX96™ Real-Time system and the cycling program consisted of an initial 95 °C for 15 min, and 40 cycles of three-step PCR at 94 °C for 15 s, 52 °C for 30 s and 72 °C for 30 s. Specificity of the reaction was checked by analysis of the melting curve of the final amplified product (from 65 to 95 °C, with increments of 0.5 °C every 0.05 s). Fluorescence was measured during the elongation step. In each reaction run, we included negative controls (without DNA) and a standard curve which consisted of four dilution points of a mixture of all cDNA samples, to calculate the reaction efficiency.

### 2.6. Quantification of Nosema Ceranae Spores

At 0, 7 and 14 days of chronic exposure to glyphosate, we collected fifteen bees per treatment (five bees per cage per three cages) and quantified *N. ceranae* spores. We removed the midguts, homogenized them individually in 1 mL of distilled water and counted the spores in a hemocytometer (Neubauer chamber improved) to estimate the number of spores per individual (intensity of parasite infection) as described on COLOSS Beebook [34,42].

### 2.7. Statistical Analyses

– Bioinformatic analysis: Illumina sequence reads were processed using R Studio Software version 4.0.2 [43] and Divisive Amplicon Denoising Algorithm 2 (DADA2) package (version 1.12.1, [44]). Low quality raw reads were discarded from obtained data and primer sequences were removed using cutadapt [45]. Then, reads were truncated to 280 bp, filtered based on length, representative sequences were obtained and denoised, and chimeric reads were removed. Then, paired reads were merged. Taxonomy was assigned to amplicon sequence variants (ASVs) using the SILVA_132 database by assignTaxonomy. Reads belonging to mitochondria, chloroplast, and eukaryotes were excluded from further analyses (“phyloseq” package version 1.28.0 [46], “subset_taxa” function). To facilitate the visualization of the barplots (relative ASVs abundance), we retained only ASVs that have at least 1% relative abundance in minimum 2 samples (“genefilter” package version 1.66.0 [47], “filterfun_sample” function). Alpha and beta diversity were calculated using the “Vegan” package [48] with the complete ASVs table. To evaluate alpha diversity, we calculated the number of observed ASVs and the Shannon index [49]. Then, we evaluated beta diversity by using Bray–Curtis, UniFrac weighted (by the relative abundance of ASVs), UniFrac unweighted and Jaccard (presence/absence of ASVs) indexes (“vegdist” function) [49]. To test the effect of treatments on community structure, we used permutational multivariate analysis of variance (ADONIS, “adonis” function) on beta diversity data. We then used the function “betadisper” to test for homogeneity of multivariate dispersions [49,50] and compared the distances of individual samples to group centroids in multidimensional space using “permutest”. The “metaMDS” function was used to plot ordinations. Then, differences between the relative abundance of different ASVs were examined using the DESeq2 software [51], as described by Jones et al. [52]. Generalized linear mixed models (GLMMs) were used to evaluate the effect of treatments (as independent variable) on alpha diversity (number of ASVs and Shannon diversity index as dependent variables) considering the cages as random effects.
– Gene expression analyses and quantification of DWV: The Ct values (threshold cycle number) of the RPS5 reference gene were used for normalization. The expression ratio between genes of interest or DWV levels and the RPS5 gene was analyzed as described by Pfaffl [53]. We used GLMMs to evaluate the effect of treatments and time on the expression of different genes or DWV levels (as dependent variables), considering the cage of origin as a random effect.
– Honey bee survival: We analyzed the effect of treatments on survival by building survival curves using the Kaplan–Meier method. Survival curves were statistically compared using the Log-rank test [54]. We then used the Cox model [55] to assess the mortality risk of the bees subjected to the different treatments.
– Food consumption: We measured daily food consumption per bee in the three cages of each treatment during the first 15 days of trials. GLMMs were used to evaluate the effect of treatments (as independent variable) on food consumption considering the time as a random effect.

In all cases, we considered *p* values below or equal to 0.05 as statistically significant. Statistical analyses were performed using R Studio Software version 4.0.2 [43].

## 3. Results

### 3.1. Impact of Glyphosate on the Gut Microbiota of Honey Bees

**Figure 1:**
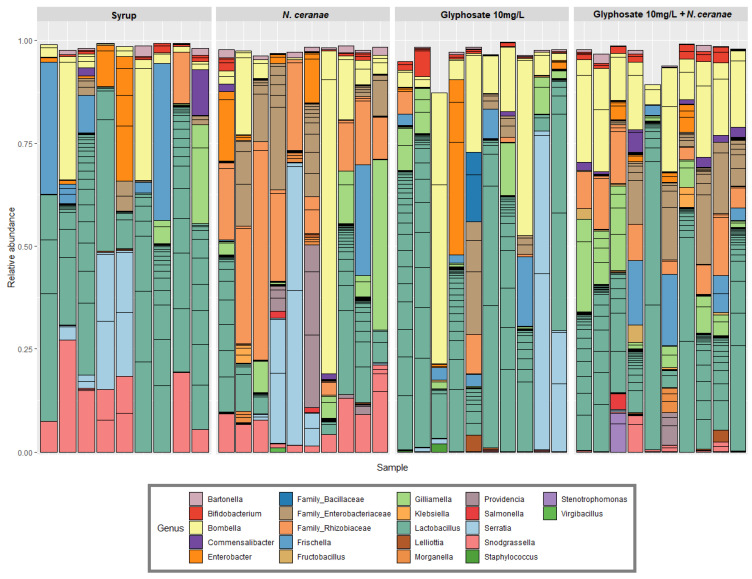
Relative abundance of different bacterial genera of the honey bee gut bacterial community subjected to seven days of chronic exposure to glyphosate in sucrose syrup (10 mg/L), *N. ceranae* infection (100,000 spores/bee at day 0) and both stress factors combined. Ten honey bees per treatment (three/four bees per cage, from three independent cages) were individually analyzed.

The impact of glyphosate alone or in combination with *N. ceranae* on the structure and composition of honey bee gut microbiota was evaluated by 16S rRNA amplicon sequencing. We obtained 3,696,421 reads, belonging to 1065 ASVs in 39 samples (with an average of 50,000 reads per sample). In all cases, the gut microbiota included the core members *Lactobacillus* spp., *Bifidobacterium* sp., *Snodgrassella* sp. and *Gilliamella* sp. among other species (Figure 1).

**Figure 2:**
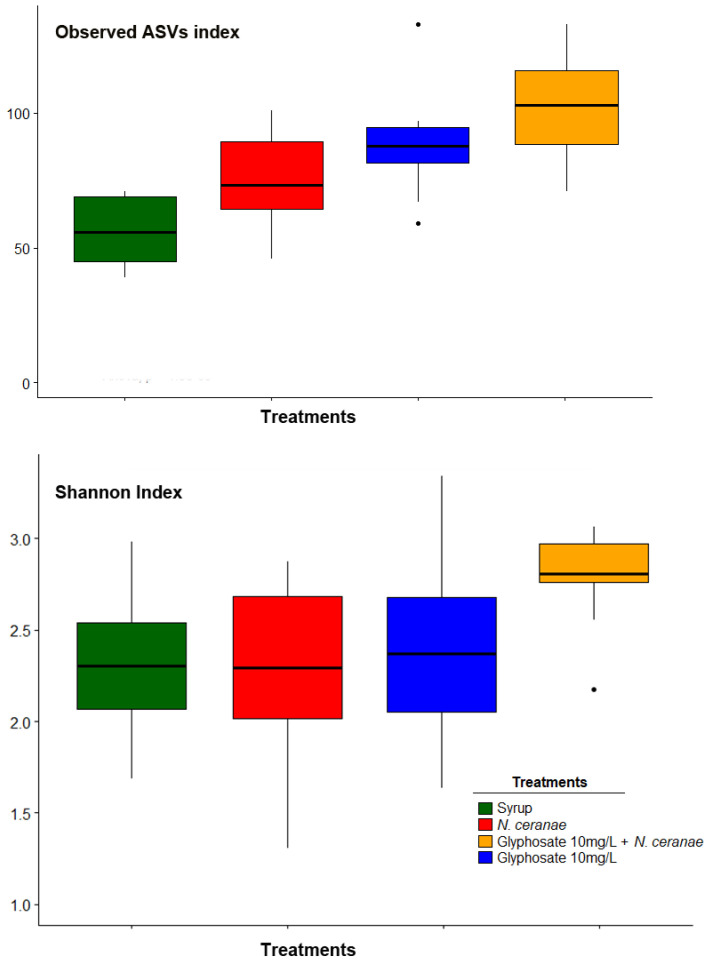
Impact of seven days of chronic exposure to glyphosate (10 mg/L), *N. ceranae* infection (100,000 spores/bee at day 0) and both factors combined on the honey bee gut microbiota: Observed amplicon sequence variants (ASVs) and Shannon Index. Ten honey bees per treatment (three/four bees per cage, from three independent cages) were individually analyzed. Results are shown as box plots, including median, 25 and 75% quartiles and outliers values.

Glyphosate, *N. ceranae,* and both stress factors together, increased the alpha diversity of the honey bee gut microbiota, as shown by the Observed ASVs index (Figure 2; Table 1). However, the Shannon index was only different when both stress factors were combined (Figure 2; Table 1).

The beta diversity metrics were also affected by the treatments. We found statistically significant differences between treatment groups independently of the distance or dissimilarity metric used (Bray–Curtis dissimilarities, weighted and unweighted UniFrac, and Jaccard index; Figure S1A–D; PERMANOVA: *p* = 0.001 in all cases). The dispersion between individuals was similar in the different groups, with the single exception of Bray–Curtis dissimilarities (Betadisper: Bray-curtis dissimilarities *p* = 0.036; Jaccard *p* = 0.06; UniFrac unweighted *p* = 0.194; UniFrac weighted *p* = 0.224).

To facilitate the visualization of the impact of both stressors, samples were divided into two groups; treated or not with glyphosate, and infected or not with *N. ceranae* (Figure S1E–J). Both stressors significantly affected the diversity of the honey bee gut microbiota (glyphosate vs. no glyphosate: Bray–Curtis dissimilarities *p* = 0.004, UniFrac weighted *p* = 0.024, UniFrac unweighted *p* = 0.001, and Jaccard *p* = 0.013; and *N. ceranae* vs. no *N. ceranae*: Bray–Curtis dissimilarities *p* = 0.001, UniFrac weighted *p* = 0.008, UniFrac unweighted *p* = 0.001, and Jaccard *p* = 0.001).

**Figure 3:**
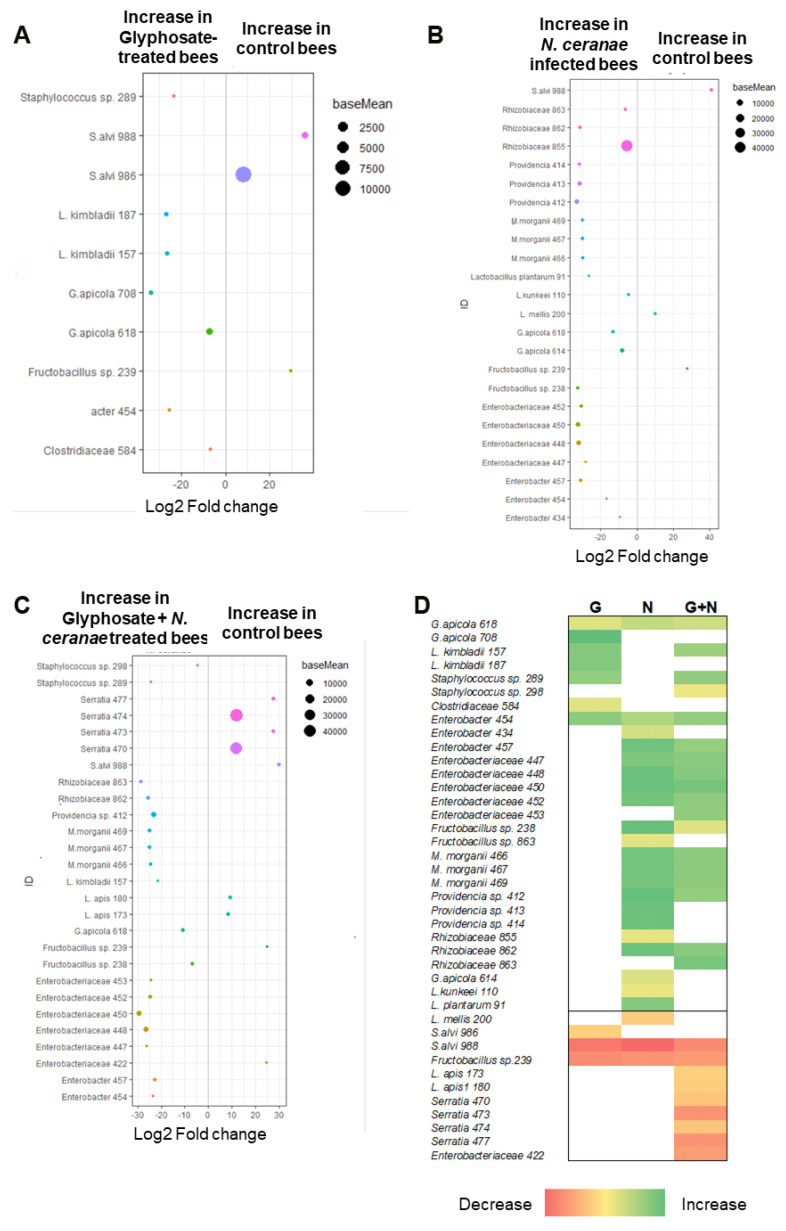
Impact of glyphosate, *N. ceranae* infection and both stress factors combined on the relative abundance of gut microbiota ASVs compared to control bees, analyzed using DESeq2. Ten honey bees (three/four bees per cage, from three cages) were individually analyzed per treatment. Only ASVs present in at least three individual samples and that show significant differences in relative abundance between groups are shown. (**A**) Comparison between honey bees subjected to chronic exposure to glyphosate (10 mg/L) for seven days and control bees; (**B**) Comparison between honey bees infected with *N. ceranae* (100,000 spores/bee at day 0) and control bees; (**C**) Comparison between honey bees subjected to both stress factors combined and control bees. In (**A**–**C**) a negative value of log2 fold changes means a decreased abundance in control bees (increased in Glyphosate/*N. ceranae* or Glyphosate + *N. ceranae* groups) and a positive value means an increased abundance in control bees (decreased in treated groups). (**D**) Summary of the results of the pair-wise comparison between treated groups and control. Green indicates an increase in treated groups and red indicate a decrease in those groups.

Using DESeq2 analysis to identify bacterial members affected by our treatments (Figure 3) we found that glyphosate alone or in combination with *N. ceranae* significantly altered the relative abundance of ASVs classified as core members, increasing the abundance of *Gilliamella apicola* and *Lactobacillus kimbladii* (*Lactobacillus* Firm-5) and decreasing the abundance of *Snodgrassella alvi,* compared to syrup-treated controls. Besides that, those treatments also increased the abundance of other species including *Staphylococcus*.

On the other hand, *N. ceranae*, alone or combined with glyphosate, increased the abundance of ASVs belonging to Enterobacterales (*Morganella*, *Providencia*, *Enterobacter* and different Enterobacteriaceae), Rhizobiaceae, *L. kunkeei*, *L. plantarum*, and *G. apicola*, compared to the syrup control, while it decreased the abundance of core member *L. mellis* and *S. alvi* among others.

Besides those changes, we observed a significant decrease in the abundance of the core member *L. apis* and the Enterobacteriaceae bacterium *Serratia* when glyphosate and *N. ceranae* were combined.

### 3.2. Impact of Glyphosate on Honey Bee Immunity

**Figure 4:**
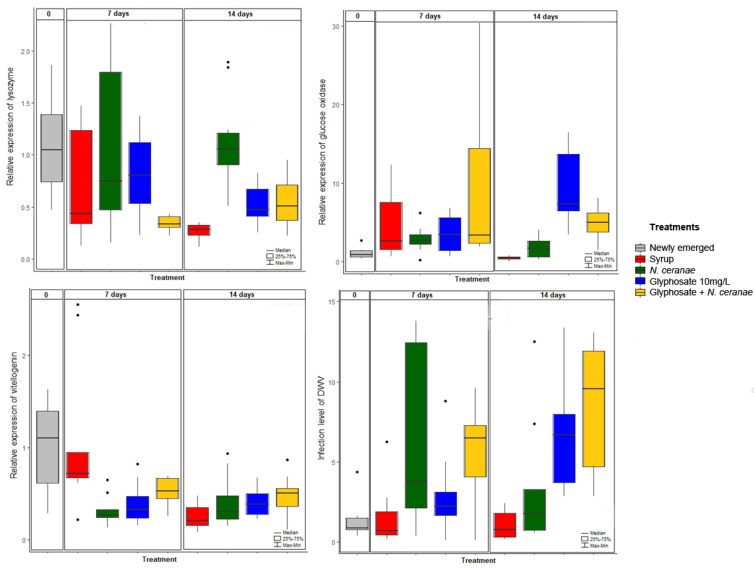
Impact of chronic exposure to glyphosate (10 mg/L), *N. ceranae* (100,000 spores/bee at day 0), and both stress factors combined on honey bee immunity (lysozyme, glucose oxidase), vitellogenin and DWV infection level evaluated using qPCR. Twelve honey bees per treatment (four bees per cage, from three independent cages) were individually sampled and analyzed at 0, 7 and 14 days of exposure. Relative quantification was performed using honey bee’s RPS5 as a reference gene. Results are shown as box plots, including median, 25 and 75% quartiles and outliers’ values.

We also tested the effect of glyphosate, *N. ceranae* or the combination of both stress factors on the expression of genes involved in honey bee immunity at different time points (Table 1, Figure 4). Glyphosate, *N. ceranae* and both stress factors combined increased the expression level of *lysozyme* compared to the control group. Glyphosate also increased the expression level of the *glucose oxidase* gene, independently of the presence of *N. ceranae*, with time of sampling having no additional impact on the expression of this gene (Table 1). On the other hand, the expression of the *prophenoloxidase* and *hymenoptaecin* genes was not affected by any of our treatments, despite these genes showing increased expression over time (Table 1). Finally, we found an effect of all the treatments, the sampling time, and of the interactions between treatments and sampling time on the expression of the *vitellogenin* gene (Table 1). In fact, while its expression decreased over time in our syrup controls (observed at day 14 in the figure), glyphosate, *N. ceranae* and their combination reduced *vitellogenin* expression already at day 7 (Table 1).

### 3.3. Impact of Glyphosate on the Dynamics of Pathogens DWV and N. ceranae

Newly emerged honey bees, and honey bees belonging to the control group at 7 days of chronic exposure to glyphosate, showed low levels of DWV infection, indicating that honey bees were naturally infected with this virus (Figure 4). Infection levels increased in bees exposed to *N. ceranae* (marginally significant difference), glyphosate or exposed to this pesticide and infected with *N. ceranae* (Figure 4, Table 1).

*N. ceranae* spores were not detected in newly emerged bees, confirming that bees were initially *N. ceranae*-free. Seven and fourteen days later, honey bees belonging to the control (syrup) and glyphosate groups remained free of spores. However, bees infected with *N. ceranae* in the absence or presence of glyphosate showed high levels of infection, reaching 3.7 × 10^6^ ± 3.6 × 10^6^ and 5.1 × 10^6^ ± 2.7 × 10^6^ spores/bee at 7 days, and 7.4 × 10^6^ ± 1.2 × 10^6^ and 6.5 × 10^6^ ± 1.5 × 10^6^ spores/bee at 14 days, respectively (Figure S2). These groups were not significantly different (G vs. GN, KW Test, *p* > 0.05 at 7 and 14 days) suggesting that glyphosate did not alter the infection by *N. ceranae*.

### 3.4. Impact of Glyphosate on the Survival of Honey Bees

**Figure 5:**
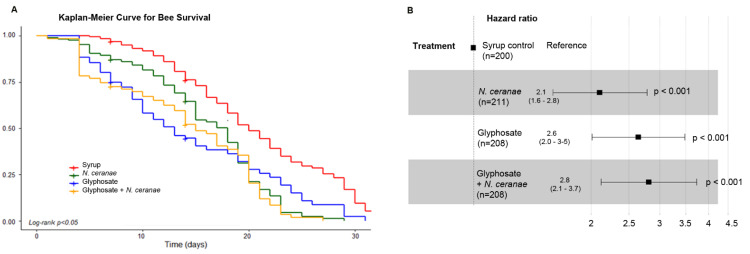
(**A**) Survival curve of honey bees subjected to chronic exposure to glyphosate (10 mg/L), *N. ceranae* (100,000 spores/bee at day 0) and both factors combined. In total, two hundred and ten bees per treatment were used (seventy bees per cage, three independent cages per treatment). (**B**) Hazard ratio of honey bees subjected to different stressors compared to the syrup control group

Glyphosate, *N. ceranae* and both stressors in combination significantly decreased honey bee lifespan (Log rank test *p* < 0.001, Figure 5A). Lethal time 50 was estimated at 20 days for control bees, 18 days for *N. ceranae* infected honey bees, 13 days for honey bees treated with glyphosate and 15 days for honey bees treated with glyphosate and *N. ceranae*. Those stressors significantly increased the risk of death by 2.1; 2.6 and 2.8-fold respectively, compared to the syrup control group (Figure 5B). It is important to notice that all bees showed similar sucrose syrup consumption/bee/day (Figure S3, Table 1).

## 4. Discussion

Glyphosate is the most widely used herbicide around the world [13], and its use continues to increase, polluting the air, rain and water courses, especially in agricultural areas [10,11]. Honey bees are exposed to this herbicide through the collection of pollen, nectar or water, and they take it to the colony contaminating other honey bees as well as their food [9,10,11]. Recent studies have shown that exposure of honey bees to glyphosate causes an alteration in the composition of the gut microbiota [15,16,17,18], which was confirmed in this study. The first published study, by Motta et al. [17] reported that the administration of glyphosate (5 and 10 mg/L) under laboratory conditions during 5 d and then returned to their colonies for three days altered the gut microbiota, being *S. alvi* the most affected species. In 2020, Motta et al. [16] confirmed that the chronic administration of this pesticide (~1.7 mg/L to 170 mg/L) to 24 h old bees under laboratory conditions affected the gut microbiota, being again *S. alvi* the most affected species. Later that year, Motta et al. [15] evaluated the oral and topical effects of various concentrations of glyphosate alone or as a commercial formulation (Round-up) under laboratory and field conditions, confirming the results previously obtained under laboratory conditions [15]. In our study, glyphosate also decreased the relative abundance of *S. alvi*, while increased the relative abundance of *G. apicola*, *L. kimbladii* (Firm-5), *Staphylococcus* sp. and Enterobacteriaceae.

The mechanism of action of glyphosate on the gut microbiota could be the inhibition of the enzyme EPSPS, which is present in the sequenced genomes of bacteria belonging to this community [17]. The susceptibility of microorganisms to glyphosate depends on the type of enzyme that they possess, class I or II. Class I enzymes are sensitive to glyphosate while class II enzymes are tolerant to the herbicide. *Snodgrassella alvi*, the species most affected by glyphosate [15,16,17,18], possesses the class I ESPS making it sensitive to the pesticide [17]. This bacterium colonizes the ileum forming a dense biofilm which could block the access of pathogens to the host epithelial cells [56]. Moreover, *S. alvi* can modulate the immune system of honey bees [57]. The decrease in the abundance of this species associated with glyphosate [15,16,17,18], reported also in this study, could thus alter the homeostasis of adult bees, having important consequences on honey bee health.

On the other hand, glyphosate increased the abundance of ASVs identified as *Staphylococcus* sp., Enterobacteraceae and core members *G. apicola* and *L. kimbladii* (Firm-5). *Staphylococcus* spp. are opportunistic microorganisms not generally found in the gut microbiota of honey bees [19,20]. However, they have been isolated occasionally from the honey bee gut, suggesting they could be part of the honey bee gut microbiota but in relatively low abundance [18,58]. Some species of the genus *Staphylococcus* have EPSPS class II, which is insensitive to glyphosate [59], so these conditions would favor the multiplication of this microorganism generating an increase in its relative abundance on honey bee gut microbiota. In fact, Blot et al. [18] showed that a *Staphylococcus* strain isolated from the honey bee gut was resistant to the herbicide.

The increase in the abundance of *G. apicola* in glyphosate-treated bees obtained in this study and previously reported [17] is difficult to interpret, since according to phylogenetic analyses using the ESPS gene they should be sensitive to glyphosate [17]. Besides that, previous studies found a reduction in its abundance associated with this pesticide [15,16,18]. Blot et al. [18] suggested that *G*. *apicola* might comprise distinct strains with different susceptibility to glyphosate, which may be related to different mechanisms of resistance, including the efflux and the enzymatic degradation of the herbicide, as it has been described in the case of other microorganisms [18,33,60].

Our results showing an increase in the abundance of *Lactobacillus* Firm-5 ASVs associated with glyphosate are consistent with previous studies [18]. Blot *et al.* [18] proposed an ecological hypothesis to explain this; the reduction of some bacterial species could release ecological niches that would be occupied by others. These ecological dynamics may explain why in our dataset *L. kumbaldi* seemed to increase when *S. alvi* abundance was reduced.

Although previously published studies provide strong evidence regarding the impact of glyphosate alone or in commercial formulations on the honey bee gut microbiota under laboratory and field conditions, the novelty of our study is the simultaneous evaluation of the impact of glyphosate together with *N. ceranae*, a microsporidium that affects bee health and is commonly found in honey bee colonies [21]. *Nosema ceranae* induced changes in the gut microbiota of honey bees, favoring an increase in abundance of bacteria in the Enterobacteriaceae family, among other alterations. Since these microsporidia infect the epithelial cells of the gut and drastically alter its anatomy and physiology [21] it is not surprising that they generate important changes in the resident gut microbiota.

Glyphosate and *N. ceranae* together increased the diversity of the gut community. Although in general a high diversity is associated with a healthy community, this does not seem the case in our study, since these stressors favored colonization by opportunistic, and potentially pathogenic, microorganisms. Besides that, both factors together decrease the relative abundance of an important core member, *L. apis* (Firm-4).

Apart from the effect of glyphosate and *N. ceranae* on the microbiota, we also investigated its impact on immunocompetence and how it affected the infection dynamics of pathogens, as well as honey bee survival. Glyphosate and *N. ceranae* increased the expression of lysozyme, an enzyme responsible for hydrolyzing cell walls in animals, and promoting the synthesis of antimicrobial peptides [61]. Glyphosate also increased the expression of glucose oxidase, an enzyme responsible for producing one of the main antibacterial components, hydrogen peroxide, by oxidizing β-d-glucose to gluconic acid in bees [62], being a marker of social immunity [63]. Those results show that glyphosate is able to modify the expression of genes associated with immunity, possibly representing a physiological response to mitigate the negative effects of poisoning [64]. On the other hand, it may be an indirect effect of the alteration of the gut microbiota. Anyway, the immune activation is energetically expensive [64] and according to our results, it was not sufficient to overcome the infection by DWV. In fact, in this study we found that glyphosate favored DWV infection, which represents the first evidence for an interaction between this pesticide and RNA viruses in the honey bee.

Glyphosate, *N. ceranae* and DWV severely decreased the expression level of vitellogenin, an important enzyme involved in division of labor, foraging specialization, queen longevity and resistance to oxidative stress [65,66]. A decrease in vitellogenin level is associated with precocious foraging and a shortened lifespan [65,66]. This decrease in vitellogenin expression may therefore be associated with the significant reduction in bee survival.

In conclusion, our results confirmed that chronic intoxication with sublethal doses of glyphosate altered the honey bee gut microbiota decreasing the abundance of core members such as *S. alvi*. When *N. ceranae* was present the effect was more evident, as gut diversity significantly increased and the abundance of other core bacteria, *L. apis,* decreased. Although glyphosate-exposed honey bees mounted an immune response (based on *lysozyme* and *glucose oxidase* genes), those strategies seemed not to be successful, as DWV infection levels increased and honey bee lifespan was significantly shortened. These findings contribute to the elucidation of the mechanisms involved in the deleterious effect of widespread glyphosate use on honey bee health.

## Figures and Tables

**Table 1:** Effect of glyphosate, *N. ceranae*, and both stress factors on honey bee gut microbiota, immunity, DWV infection level and syrup consumption, evaluated by generalized linear mixed models. *p* values under 0.05 were considered statistically significant and are shown in bold. * indicates interaction between factors.

Dependent Variable	Independent Variable	Coefficient Value	Intercept Value	*p* Value
Observed ASVs	*N. ceranae*	0.2	4.01	**0.004**
	Glyphosate	0.46		**≤0.001**
	*N. ceranae* + Glyphosate	0.62		**≤0.001**
Shannon diversity index	*N. ceranae*	−0.005	0.83	0.95
	Glyphosate	0.01		0.86
	*N. ceranae* + Glyphosate	0.19		**0.03**
Lysozyme	*N. ceranae*	1.38	−1.29	**≤0.001**
	Glyphosate	0.69		**≤0.001**
	*N. ceranae* + Glyphosate	0.67		**≤0.001**
	Time	-		ns
Glucose oxidase	*N. ceranae*	0.07	−0.24	0.16
	Glyphosate	0.32		**≤0.001**
	*N. ceranae* + Glyphosate	0.25		**≤0.001**
	Time	-		ns
Prophenol oxidase	*N. ceranae*	0.04	−0.37	0.24
	Glyphosate	−0.007		0.83
	*N. ceranae* + Glyphosate	0.04		0.17
	Time	0.15		**≤0.001**
Hymenoptaecin	*N. ceranae*	0.77	0.99	0.05
	Glyphosate	0.24		0.57
	*N. ceranae* + Glyphosate	0.33		0.43
	Time	1.67		**≤0.001**
Vitellogenin	*N. ceranae*	−0.16	−0.16	**≤0.001**
	Glyphosate	−0.14		**≤0.001**
	*N. ceranae* + Glyphosate	−0.12		**≤0.001**
	Time	−0.08		**≤0.001**
	N. ceranae * Time	0.09		**≤0.001**
	Glyphosate * Time	0.08		**≤0.001**
	*N. ceranae +* Glyphosate * Time	0.07		**≤0.001**
Deformed Wing Virus	*N. ceranae*	0.44	−0.24	0.07
	Glyphosate	−0.11		**0.01**
	*N. ceranae* + Glyphosate	0.03		**≤0.001**
	Time	-		ns
Syrup consumption	*N. ceranae*	−0.02	2.65	0.73
	Glyphosate	−0.07		0.28
	*N. ceranae* + Glyphosate	0.08		0.16

## Data Availability

The sequencing data have been deposited with links to BioProject accession number PRJNA719169 in the NCBI BioProject database (https://www.ncbi.nlm.nih.gov/bioproject/).

## Supplementary Materials

The following are available online at https://www.mdpi.com/article/10.3390/microorganisms9040845/s1.
