# Supplementary material for: Impact of Chronic Exposure to Sublethal Doses of Glyphosate on Honey Bee Immunity, Gut Microbiota and Infection by Pathogens
Source: Microorganisms. 2021 Apr 15;9(4):845. doi: 10.3390/microorganisms9040845 (PMC8071123; doi:10.3390/microorganisms9040845)
Supplement: Supplementary file 1 [file microorganisms-09-00845-s001.zip › microorganisms-1165486-supplementary.pdf]

Supplementary material

Impact of chronic exposure to sublethal doses of glyphosate on honey bee immunity, gut microbiota and infection by pathogens

Loreley Castelli[1], Sofía Balbuena[1], Belén Branchiccela[2], Pablo Zunino[1], Joanito Liberti[3,4], Philipp Engel[3], Karina Antúnez[1]*.

[1] Departamento de Microbiología, Instituto de Investigaciones Biológicas Clemente Estable. Montevideo, Uruguay.
[2] Sección Apicultura, Instituto Nacional de Investigación Agropecuaria. Colonia, Uruguay.
[3] Department of Fundamental Microbiology, University of Lausanne, Lausanne, Switzerland
[4] Department of Ecology and Evolution, University of Lausanne, Lausanne, Switzerland

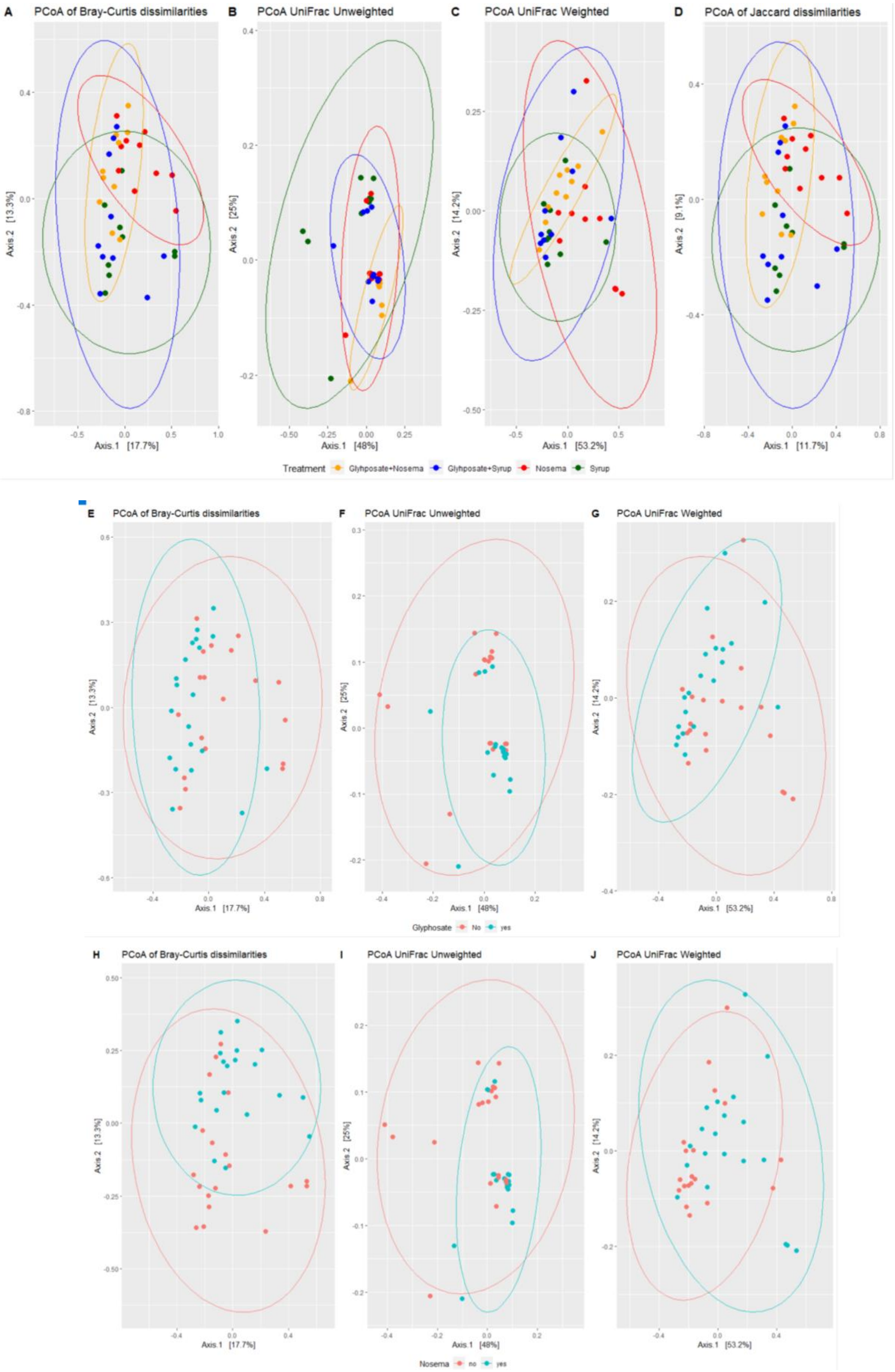

Figure S1.- Impact of chronic exposure to glyphosate (10 mg/L), *N. ceranae* (100.000 spores/bee at day 0) and both stress factors on honey bee gut microbiota. Beta diversity analyses showed that the treatments altered the structure and membership of the gut microbiota based on Bray-curtis dissimilarities, weighted and unweighted UniFrac and Jaccard indexes (p= 0,001 in all cases, A-D). Samples were divided into two groups; treated or not with glyphosate (E-G), and infected or non with *N. ceranae* (H-J). Both stressors significantly affected honey bee microbiota diversity ($p<0.05$ in all cases).

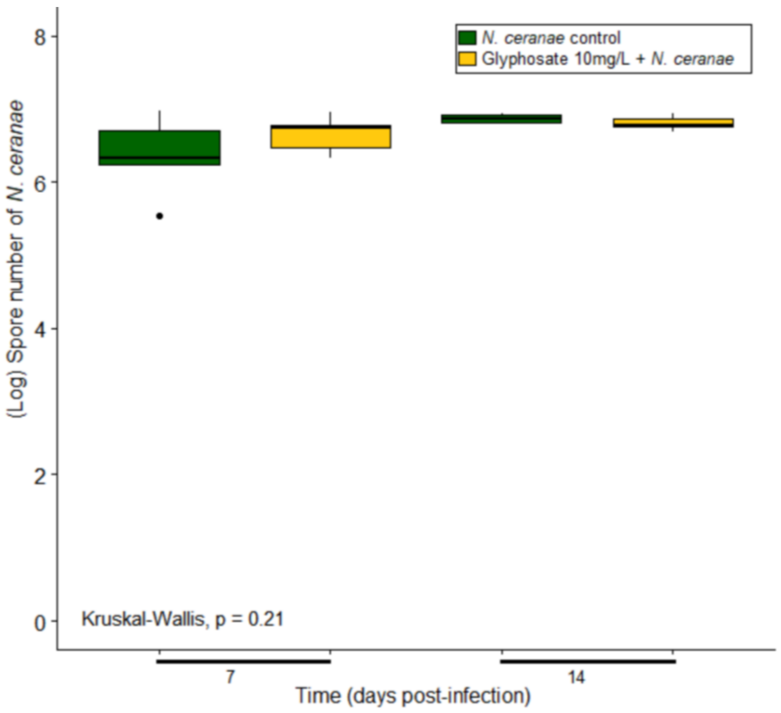

Figure S2.- *Nosema ceranae* spores number per bee on honey bees artificially infected with 100.000 spores, and on bees infected and exposed to glyphosate (10 mg/L) 7 and 14 days post-infection. No significant differences were found between those groups. *N. ceranae* spores were not detected in newly emerged bees, or honey bees belonging to the control (syrup) and glyphosate groups.

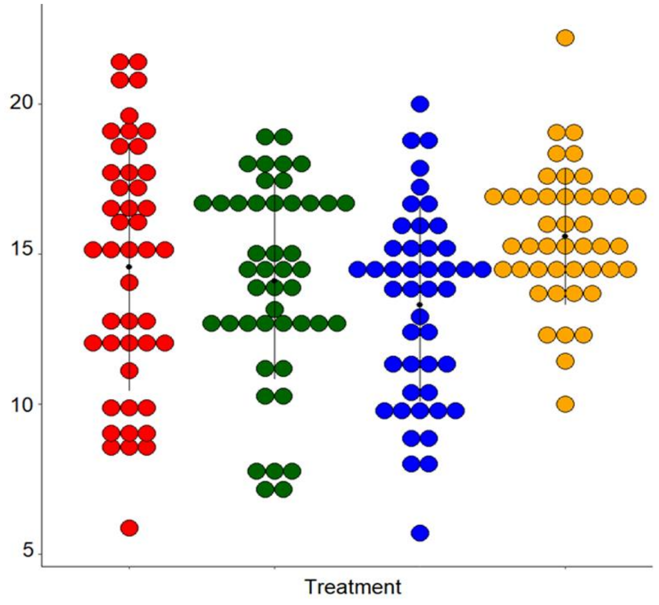

Figure S3.- Sucrose syrup consumption (μl) per bee per day during the first 15 days of experiment in honey bees subjected to different treatments: syrup control (red), *Nosema ceranae* spores (green), glyphosate (10 mg/L, blue) and both stress factors combined (yellow). No significant differences in sugar consumption was observed between groups.
